# Supplementary material for: Methane-Mediated Vapor Transport Growth of Monolayer WSe2 Crystals
Source: Nanomaterials (Basel). 2019 Nov 19;9(11):1642. doi: 10.3390/nano9111642 (PMC6915445; doi:10.3390/nano9111642)
Supplement: Supplementary file 1 [file nanomaterials-09-01642-s001.pdf]

Supplementary Material

Methane-Mediated Vapor Transport Growth of Monolayer WSe_2 Crystals

Hyeon-Sik Jang [1,†], Jae-Young Lim [1,†], Seog-Gyun Kang [1], Sang-Hwa Hyun [2], Sana Sandhu [1], Seok-Kyun Son [3], Jae-Hyun Lee [2,*] and Dongmok Whang [1,*]

[1] School of Advanced Materials Science and Engineering and SKKU Advanced Institute of Nanotechnology (SAINT), Sungkyunkwan University (SKKU), 2066, Seobu-Ro, Jangan-Gu, Suwon-Si, Gyeonggi-Do 16419, Korea; dagu1821@skku.edu (H.-S.J.); limjyyy@skku.edu (J.-Y.L.); suggyoon@skku.edu (S.-G.K.); sanasandhu_7@yahoo.co.in (S.S.)

[2] Department of Energy Systems Research and Department of Materials Science and Engineering, Ajou University, Suwon, Gyeonggi-Do 16499, Korea; ruche@ajou.ac.kr

[3] Department of Physics, Mokpo National University, Muan-gun, Jeollanam-Do 58554, Korea; skson@mokpo.ac.kr

* Correspondence: jaehyunlee@ajou.ac.kr (J.-H.L.); dwhang@skku.edu (D.W.); Tel.: +82-31-210-2465 (J.-H.L.); +82-31-290-7399 (D.W.)

† These authors contributed equally to this work.

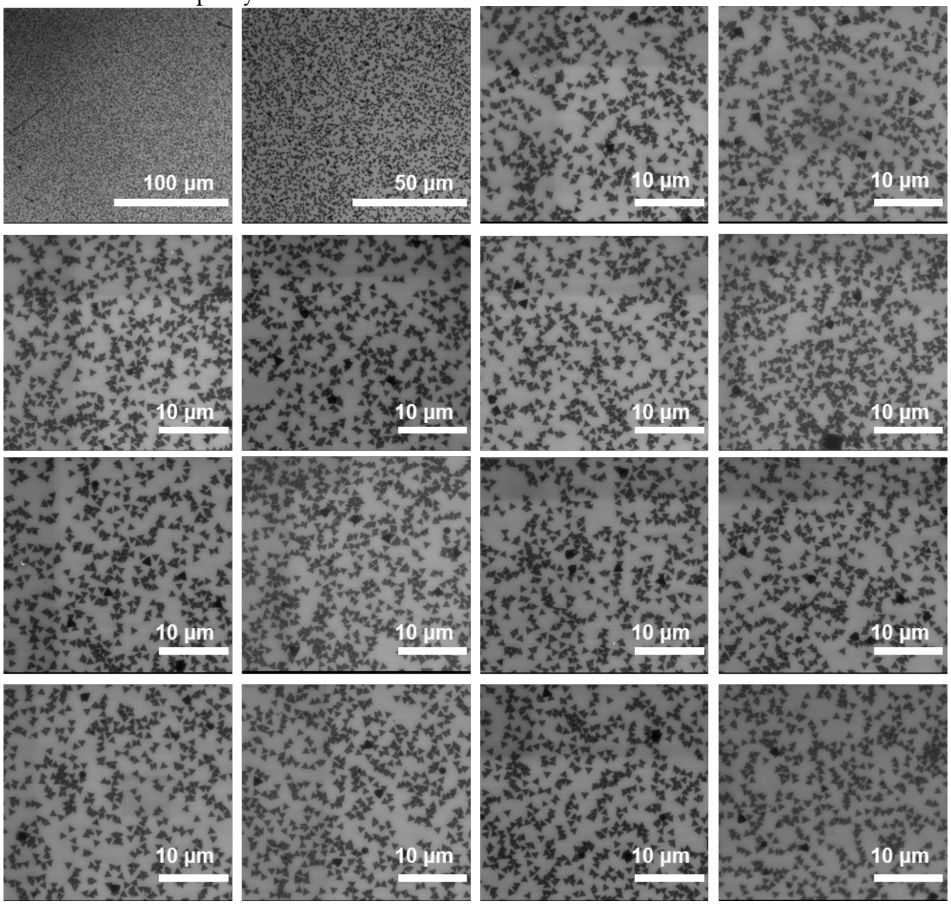

Figure S1. SEM images of synthesized WSe_2, which were used for analysis of the nucleation density and edge length of WSe_2 domains. Carrier gas ratio was CH_4 : Ar = 0 : 200 sccm

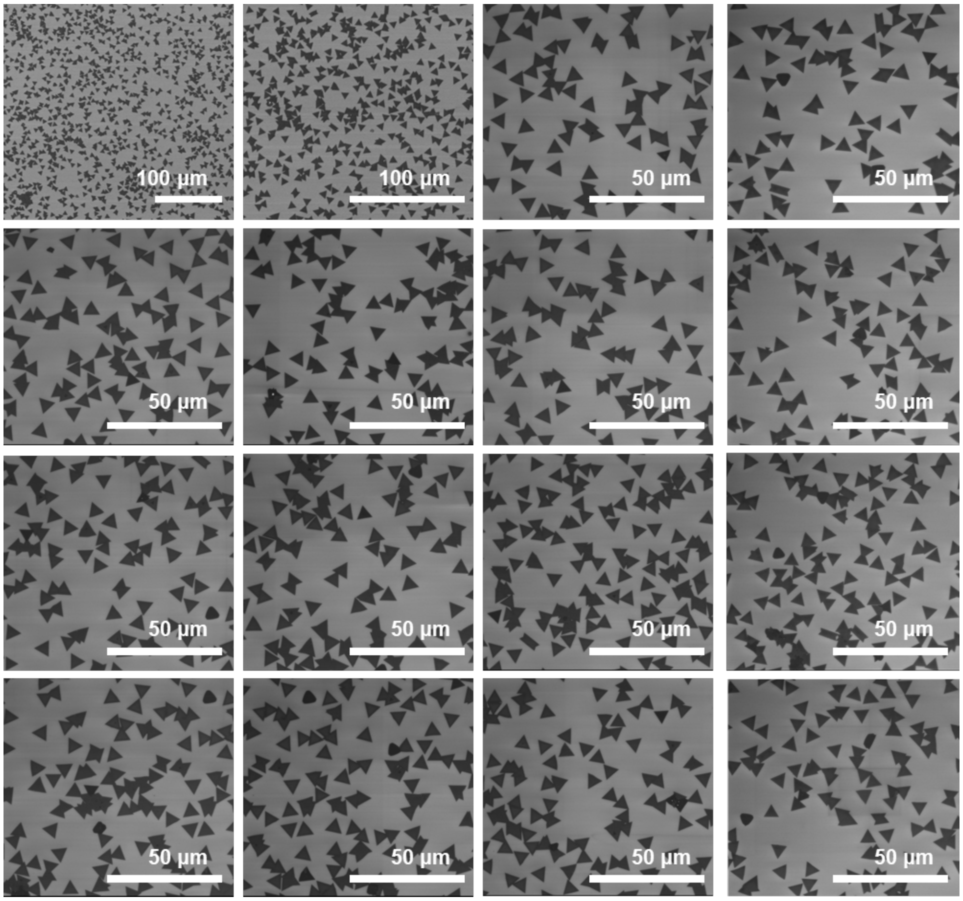

Figure S2. SEM images of WSe_2 domains synthesized with carrier gas ratio of CH_4 : Ar = 50 : 150 sccm.

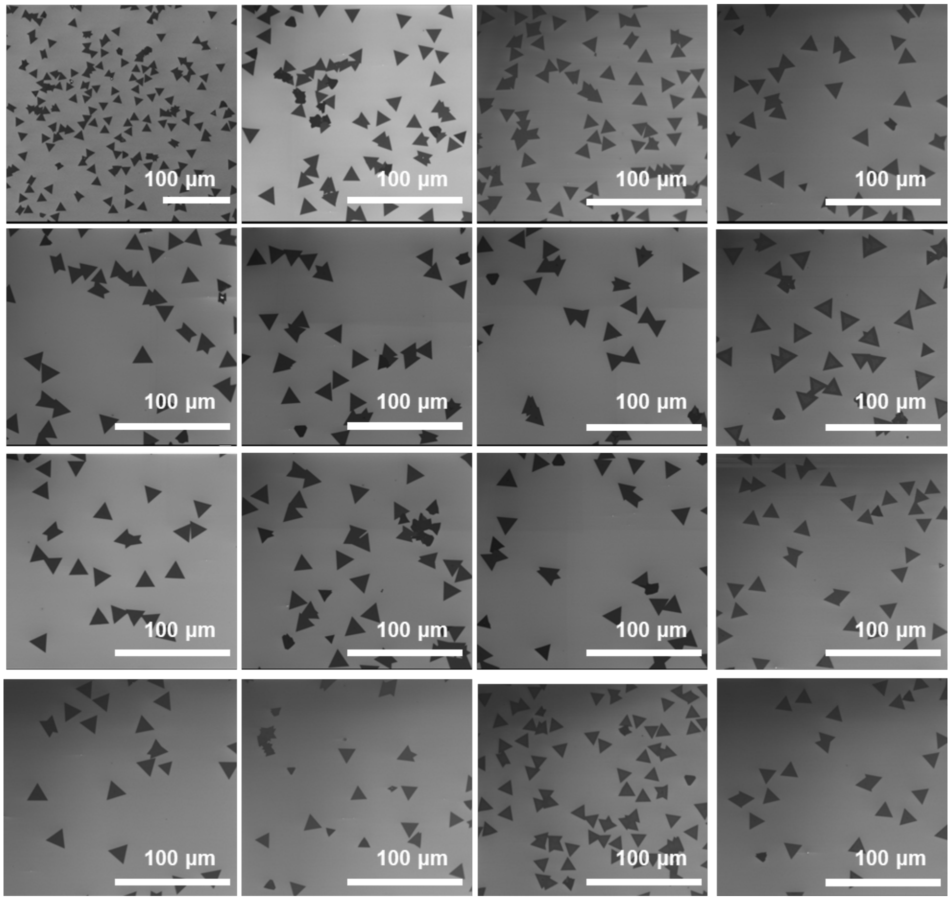

Figure S3. SEM images of WSe_2 domains synthesized with carrier gas ratio of CH_4 : Ar = 100 : 100 sccm

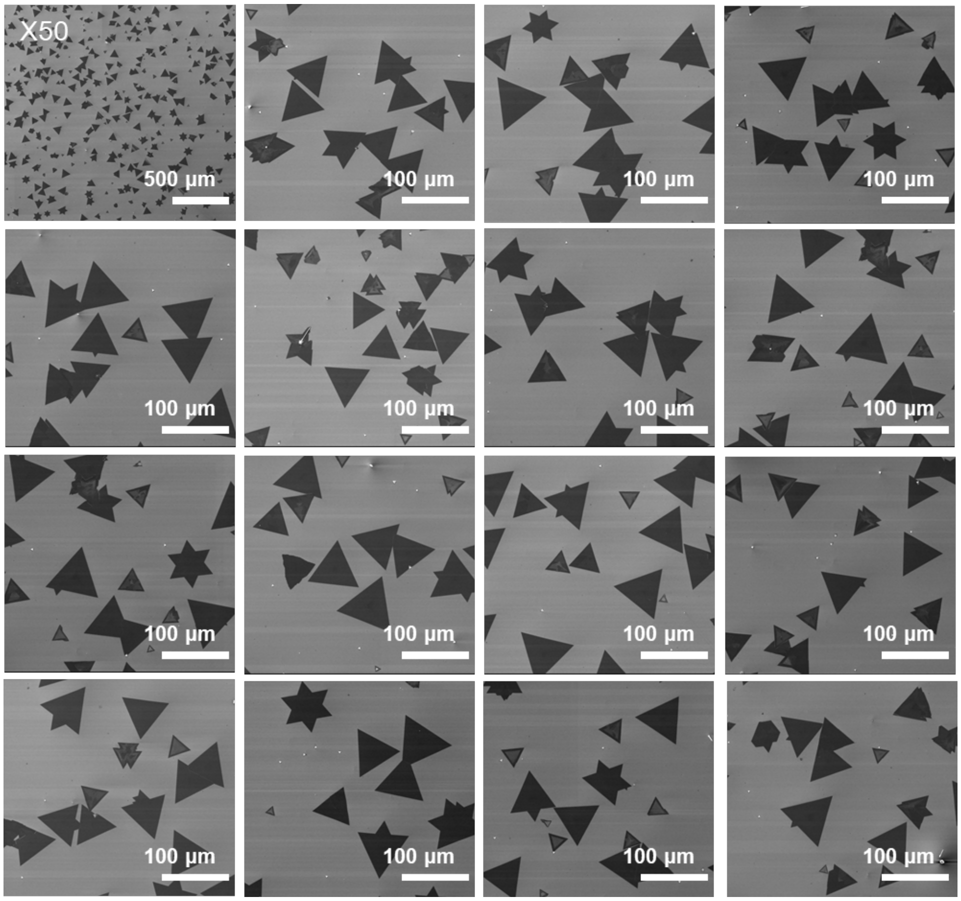

Figure S4. SEM images of WSe_2 domains synthesized with carrier gas ratio of CH_4 : Ar = 150 : 50 sccm

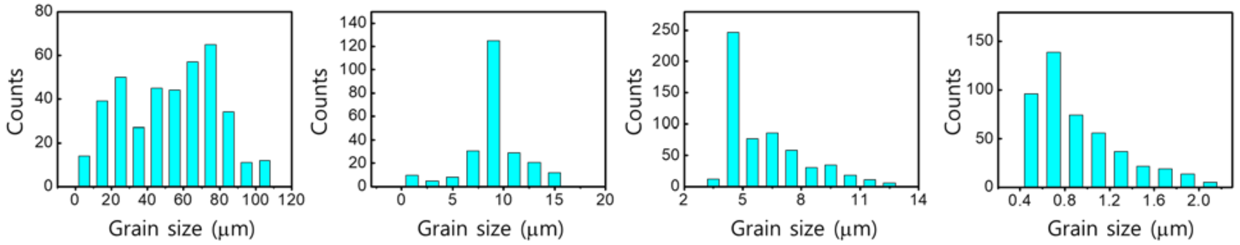

CH$_4$: Ar (sccm)	150 : 50	100 : 100	50 : 150	0 : 200
Average size (mm)	52.67737	8.97221	6.13638	0.92437
Standard deviation	25.72943	2.8419	1.97363	0.38015

Figure S5. Distribution of grain sizes in WSe_2 according to carrier gas ratios.

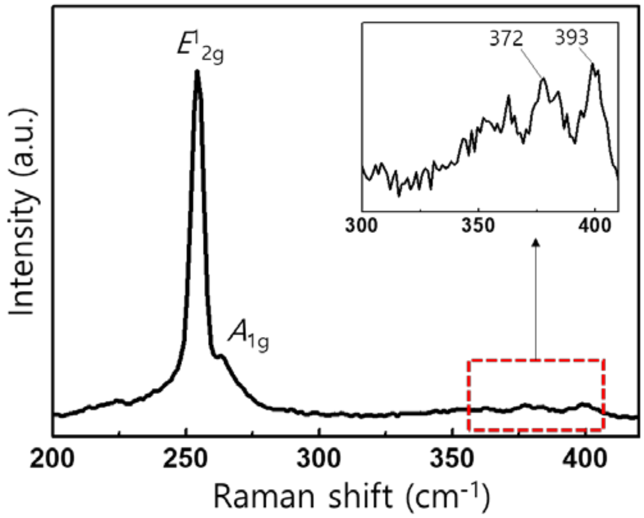

Figure S6. Raman spectrum of synthesized monolayer WSe_2.

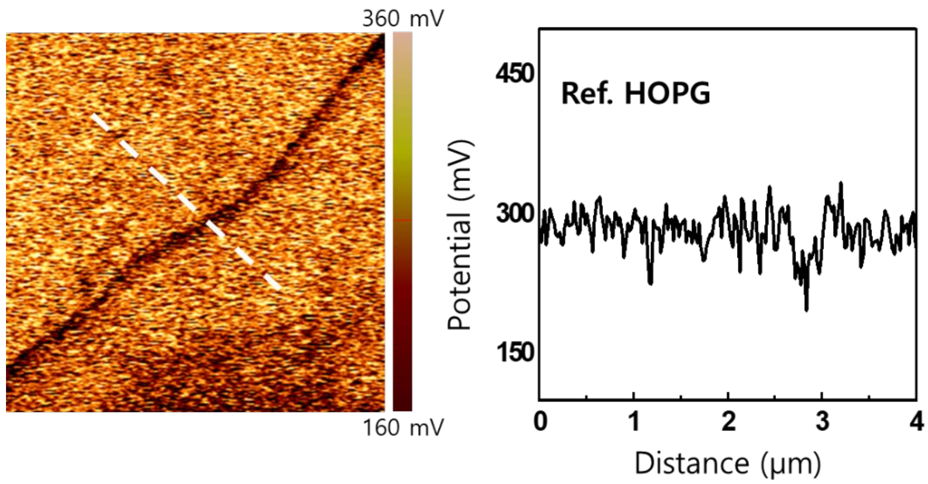

Figure S7. The work function of Pt coated AFM tip and reference HOPG

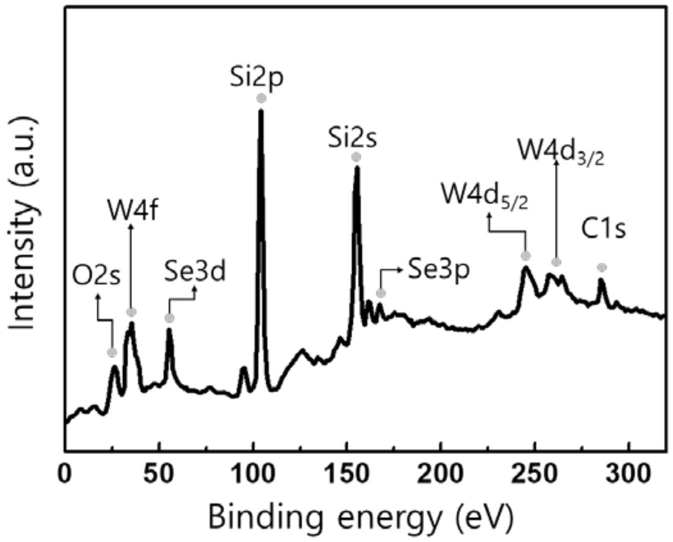

Figure S8. XPS wide-range spectra of synthesized WSe_2
